# Supplementary material for: As time goes by: Long-term retention of economics skills
Source: PLoS One. 2025 Dec 12;20(12):e0333305. doi: 10.1371/journal.pone.0333305 (PMC12700375; doi:10.1371/journal.pone.0333305)
Supplement: Attachment — Submitted filename: response-to-reviewers.pdf [file pone.0333305.s001.pdf]

Response to Reviewers

We sincerely thank the reviewers and the editor for their thoughtful feedback. Below, we address each comment point by point. All changes made in the manuscript are highlighted and referenced accordingly.

Reviewer 1

1. *The literature review seems to not consider economics articles on retention. Juttler (2020, Feb) in this journal or Cosgrove and Olitsky (SEJ, 2015) which although for high school is still relevant.*

   Juttler (2020) looks at a very different type of retention: Their paper analyzes the determinants of Swiss high school students' decisions to continue their study of economics five years later. We have revised the introduction of our paper to note that the word "retention" has two distinct uses in economic education research, and that our paper studies knowledge retention as opposed to retention of students in the major.

   Cosgrove and Olitsky (2015) study students' learning and knowledge retention in two introductory economics courses taught over multiple academic terms using various teaching methods. Students are given the same set of 10 multiple choice questions on the first day of class (before the material is taught), on the ninth day of class (after the material is taught), and on the last day of class. The authors compare the second test to the first test to measure initial learning, and use the difference between the third test and the second test as a measure of knowledge retention. They find that on average, student knowledge declines between the second test and the end of the term. Our paper looks at change in student knowledge between the end of the term and points one to 2.5 years later. We have incorporated this article into our review of the literature, but we have not found any other papers in economics that study knowledge retention.

2. *The next to last paragraph says that a limitation of other studies is not considering the role of some characteristics. You say this is helpful. Expand on why.*

   We have expanded this paragraph to better explain why this is helpful:

   > One limitation shared by all these studies is that none empirically examined the potential mediating role of student characteristics, relevant coursework completed, or work experience acquired within the retention interval. These factors might hold a great deal of information that could be useful for educators in designing their course plans. *That is, if some groups systematically retain less of what we teach, instructors could pay particular attention to those groups to instill a deeper level of understanding. Similarly, instructors should consider encouraging post-course behavior associated with higher retention of course skills.* Our paper addresses this important limitation.

3. *Target sample is misleading. Isn't the entire class the target? The description of the samples was difficult to follow.*

   Because only a subset of the students in each original course took the end-of-term assessment and consented to be part of the research study, we only targeted these students for follow-up. Even if we could have surveyed the other students in the course, we would not have measures of their skills at the end of the term to compare to follow-up measures. This limits our ability to generalize our results to population of students enrolled in each class, but Table 1 shows that the distribution of students by college year and the mean final exam scores for all enrolled students are quite similar to those of the students we targeted for follow-up. In three of the cohorts there are no significant differences, and in the other three, final exam scores in the target samples are 1.4-2.2 points higher and freshman are slightly over-represented.

   We have revised the text to more clearly define the target samples and how they differ for the broader set of students enrolled in each course.

4. *Do you know how much time they spent the first time the completed the assessment?*

   We use the measure of time spent on the initial (end-of-term) online assessment as a proxy for student effort on the online assessment in order to check the validity of submitted answers. We drop students who spent 10 minutes or less from our analysis samples. This was between 6 and 22% of the students who completed PESA-Micro in our introductory microeconomics samples, none of the students in the first two cohorts of intermediate microeconomics where exam was given in-person, and 7% of the students in the last (Fall 2020) cohort of intermediate microeconomics. While calculating these numbers, we discovered that we had mistakenly included a small number of low effort students in our analysis—Our samples and results have been corrected in the revised draft.

5. *Better explain the normalization. what are the new mean and s.d.?*

   All scores that enter our models are standardized using the distribution from the original cohort's baseline distribution to simplify interpretation and comparison of coefficients. That is, we subtract the mean score in the original cohort and divide by its standard deviation yielding baseline scores that are mean zero and unit variance. We have revised the text to make this clearer.

6. *You say MESA scores declined substantially. Substantial is subjective. The percentage decline suggests a 1.8 item decline on average. My interpretation is that is substantial retention.*

   The reviewer makes an excellent point, and we have revised our text accordingly:

   > We can also see in these tables that PESA scores on average declined between the end of the course and both follow-ups for all three cohorts, though students retained most of what they knew, and declines were modest. In the Fall 2022 cohort there was only a 1-point decline by the end of the first year, while scores decline by 6 points at the one year follow up for the Fall 2019 cohort and by 3 points for the Fall 2020 cohort. At the two-year follow-up, declines from baseline were almost the same as those observed at the one year follow-up.
   >
   > We observe declines in scores on end-of-term tests during the follow-up period that were somewhat larger than we observed for the introductory courses, though there is still substantial retention. The average decline for the Spring 2019 cohort was

7 points at 1.5 years and 11 points at 2.5 years, and the declines observed for the Fall 2019 cohort were 11 points at 1.5 years and 9 points at 2.5 years. Scores of students in the Fall 2020 cohort declined by 7 points and 8 points at the 1.5-year and 2.5-year follow-ups respectively.

7. *Is it retention? Suppose only a 3 item quiz and initially the student gets 2 of 3 correct. On the retake they get 1 of 3 correct, but the one they got correct is the one they initially got wrong. You would say they retained half of what the initially knew, but in fact they retained nothing.*

   The reviewer is right that changes in performance scores are an imperfect measure of knowledge retention. We have added the following text to the beginning of our Methods section:

   > Note that the change in assessment score is not a perfect measure of knowledge retention. For example, a student's score could remain the same at the follow-up if they forget some course concepts they knew at the end of the course but also learn course concepts they had not learned during the course. In this case, the change in score would over-estimate the amount of knowledge that is actually retained by the student.

   While we continue to use changes in scores as a proxy for knowledge retention in this paper, we are working on a separate paper that distinguishes learning, forgetting, and retention by analyzing assessment questions individually. We briefly discuss this work in our conclusion. In our preliminary results we find there is little evidence of significant learning during the follow-up periods. That is, students tend to remember much of what they knew at the end of the term, and continue to incorrectly answer the same questions they incorrectly answered at the end of the term.

8. *Is IESA asking about MRS and MRTS? Should we expect students to remember that? In micro you learn of sunk costs, that can come up in life (same with supply and demand).*

   The reviewer raises an important general point. Some questions might easier for former students to answer because they do not rely on economic jargon or because they require specific calculations. Given that intermediate microeconomics is a more technical course, this could explain why observed knowledge retention is lower. As noted in the conclusion of the paper, we are working on a follow-up paper that explores differences in retention by types of questions where type is defined by difficulty, complexity, and how much calculation is required.

   To answer the reviewer's specific question, IESA does not ask students to give definitions of MRS or MRTS, but it does contain two questions that use the concept of Marginal Rate of Substitution. Question 3 asks students to compute the marginal cost of one good in terms of the other, and Question 4 asks students to identify the shape of an indifference curve given the MRS, though it is not explicitly called the MRS:

   > IESA Q3. Suppose an individual consumes only two goods, x and y. Given her fixed income and prices of the goods, the consumer can purchase 5 units of x and no units of y *or* 3 units of y and no units of x *or* any linear combination of these two extremes. The consumer's marginal opportunity cost of one unit of y ________ .
   >
   > (a) is $\frac{3}{5}$ units of x

(b) is $1\frac{2}{3}$ units of x
(c) is 5 units of x
(d) is 15 units of x
(e) cannot be determined, given the information above

IESA Q4. Suppose a consumer's preferences are described by a utility function $u(x, y)$, such that the ratio of marginal utilities is given by

$$\frac{MU_x}{MU_y} = \frac{3x}{8y},$$

The shape of an indifference curve for this consumer is best described by

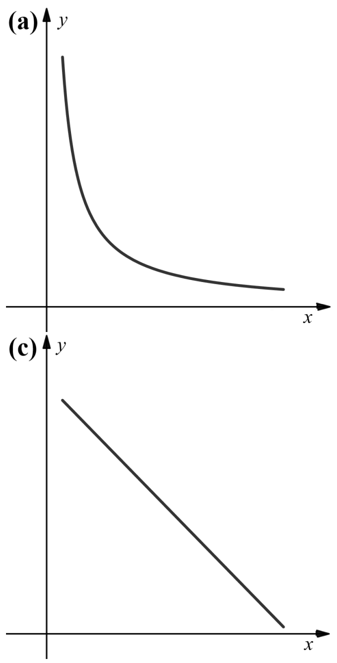

9. *Do you have enough observations to report a regression for Spring 2023 of the intermediate sample?*

   We currently pool data from the first follow-up of all three intermediate micro cohorts for our analysis. We do the same for the second follow-ups. This approach yields 136 observations for the first follow-up and 89 for the second. Unfortunately, the sample sizes for the individual cohorts are quite small: 54 for the first follow-up of the Fall 2020 sample and 37 for its second follow-up (in Spring 2023). We do not consider these large enough to support a useful regression analysis.

10. *The entire discussion section is your unsubstantiated opinion of why results look that way. For example, you have no idea why female students have different scores. I would argue for deleting this section.*

    The reviewer is correct that we do not have strong evidence of the mechanisms that explain the gender differences in retention that we observe, and we have removed the paragraph

speculating about potential mechanisms from our discussion section. However, we feel it is important to raise potential explanations grounded in theory for some other our other findings in hopes that future research will provide empirical evidence. We have tried to make this rationale clearer in the paper, but we are happy to defer to the editor if they would prefer us to remove this section entirely.

Reviewer 2

1. *P3: line 77-79: Be explicit about how the differences in course teaching method and modality are captured in the cohort fixed effect*

   We have revised the paper and included the following paragraph at the beginning of the Methods section:

   > While the instructor and content of all three cohorts of each course were the same, there were differences in how these courses were taught. Specifically, some courses were taught online during the COVID-19 pandemic, while others were taught in-person before or after the pandemic. Similarly, the instructors spent different fractions of class time lecturing and having students engage in problem solving activities in the different academic terms. We estimate our regression models using pooled data from all the cohorts for a given course, allowing us to maximize our sample size while still including cohort fixed effects to account for unobserved differences between cohorts. That is, our models allow the average change in assessment scores to differ by cohort, holding student characteristics constant.

   In a separate paper, we look closely at the impact of these differences in pedagogy on retention of skills.

2. *P3: line 89 and P7: line 180: Can you say more about why the reader should be confident that 30 (or 31 for intermediate) multiple choice questions are a reasonable measure of student learning?*

   While no assessment can be a complete measure of learning in a course, economics and other STEM disciplines have a strong tradition of using well-designed multiple choice tests as proxies for course learning. These include the Test of Understanding College Economics (Walstad et al., 2007), the Force Concept Inventory (Hestenes et al., 1992), the Genetics Concept Assessment (Smith et al., 2008), and the Chemical Concept Inventory (Mulford and Robinson, 2002). Both assessments used in this paper (PESA-Micro and IESA-Micro) were developed using the best practices outlined in Adams and Wieman (2011). Each assessment question maps to specific learning goals of the relevant course to ensure broad coverage, and the questions were developed using an iterative process that included student think-aloud interviews, faculty feedback, and pilot data. As further evidence that our assessments are reasonable measures of student learning, we find that they are strongly correlated with final exam scores of students. These correlations are 0.53, 0.45, and 0.20 for the three cohort samples of introductory microeconomics, and 0.45, 0.56, 0.33 for the cohort samples of intermediate microeconomics. We have incorporated this information into the Methods section of the revised paper.

3. *P3: line 99 (and equivalent for intermediate): What can you say about the external validity of the findings (beyond your own institution)?*

We originally addressed this very briefly in our Conclusion, and we have expanded this discussion with the following paragraph:

> A related concern is external validity. Our analysis sample is determined by the data collection design and is limited to data from six terms of two courses taught by two instructors at one institution. While Cornell University is not representative of all universities, most economics departments around the world offer courses with content that is very similar to these courses. In addition, Cornell is a selective R1 university with a student body very much like other selective R1 universities in the US. Future research could replicate this work at other institutions, with other instructors, in other courses, or even in other disciplines to determine whether long-term retention of knowledge and skills operates similarly in different contexts.

4. *P6: lines 124-126: Provide further explanation about how information on "courses that used their economic knowledge" and jobs where "they applied their economic knowledge" was gathered. Was this based on the student's own interpretation of what using/applying economic knowledge meant? Was economic knowledge defined, or confined specifically to microeconomic knowledge? The specifics of the additional course might matter – e.g. upper level applied micro course.*

   We have clarified how these variables were constructed in our revised draft: $\mathbf{X}_{i,t}^{\text{behavior}}$ is a vector of variables representing work and course enrollment since the end of the course or the previous follow-up. These variables are constructed from answers to the following three yes/no questions given to participants during the follow-up survey:

   (a) Have you completed any courses where you extended or applied your knowledge of microeconomics since [DATE OF END OF COURSE IF FIRST FOLLOW-UP or DATE OF FIRST FOLLOW-UP IF SECOND FOLLOW-UP]?

   (b) Are you currently enrolled in any courses where you are extending or applying your knowledge of microeconomics?

   (c) Since you took introductory microeconomics, have you worked in any job where you used your microeconomics knowledge?

   In some models, we use binary indicators based on answers to each of the above questions, and in some specifications we combine the two course measures into one that measures whether they were currently enrolled in a course that used their economics knowledge or if they completed at least one since the end of the course.

In addition to the questions above, the survey asked students to list the relevant courses the took or were currently enrolled in. We invested substantial time in coding these courses as introductory, intermediate, or advanced using their course numbers. However, neither level of course nor the quantity of courses taken made a significant difference in our models of retention. This lack of significance is likely attributable, at least in part, to the limited sample size, which made it difficult to detect meaningful differences.

The results are presented separately for intro and intermediate micro, implying that these samples are separate. Might some of the students in the (earlier) intro cohorts also be included in the intermediate sample? If so, does that/how might that matter?

Our six cohorts contain a total of 1,022 students. Only 11 of these students appear in both an introductory and an intermediate cohort. This is in large part because of the timing of the study

and the structure of the economics curriculum at Cornell. Two of the intermediate cohorts were taught before or simultaneous with the earliest introductory cohort. The third intermediate cohort was taught a year after the first introductory cohort (Fall 2019), simultaneous with the second, and after the third. While introductory microeconomics is a prerequisite for intermediate, the third intermediate cohort drew students from several introductory courses in addition to the Fall 2019 target course.

We make no corrections in our analysis for these 11 students because there are so few and our analyses of knowledge retention in introductory and intermediate microeconomics are separate studies with very similar structure. When we analyze these students as introductory microeconomics students, their experience in intermediate microeconomics is incorporated through our course enrollment variables. These students were asked to complete follow-up assessments for both courses, but we do not believe answering these questions during a follow-up survey is a sufficiently strong learning experience to influence our results.

*P10: In discussing the results, how do you distinguish between retention of learning from intro micro and *re-learning* concepts in another economics course?*

Reviewer 1 also raised this important issue, and both reviewers are right that changes in performance scores are an imperfect measure of knowledge retention. We have added the following text to the beginning of our Methods section:

> Note that the change in assessment score is not a perfect measure of knowledge retention. For example, a student's score could remain the same at the follow-up if they forget some course concepts they knew at the end of the course but also learn course concepts they had not learned during the course. In this case, the change in score would over-estimate the amount of knowledge that is actually retained by the student.

While we continue to use changes in scores as a proxy for knowledge retention in this paper, we are working on a separate paper that distinguishes learning, forgetting, and retention by analyzing assessment questions individually. We briefly discuss this work in our conclusion. In our preliminary results we find there is little evidence of significant learning during the follow-up periods. That is, students tend to remember much of what they knew at the end of the term, and continue to incorrectly answer the same questions they answered incorrectly at the end of the term.

P13: Line 320 and 325: Do you have evidence to support the purported gender difference in study habits? If so, provide detail. If not, it would be better to remove purely speculative material.

The reviewer is right to point out this is an unsubstantiated theory. We have removed this paragraph from the paper.

P13: Line 342: This depends on what is being captured in the "jobs that apply economics concepts". (See earlier comment about defining this more fully.)

We agree completely with the reviewer, and we believe the precise definition of this measure earlier in the revised paper makes this part of the discussion much clearer.

Some of these questions are likely not answerable in a definitive way: the complexities in interpreting the results should be explicitly acknowledged.

We agree, and have made three changes to the paper to reflect this. First, we have more precisely defined the variables related to interim course and job experience. Second, after we define these variables, we have added the following paragraph:

> While all respondents who answer these questions affirmatively believe they are either applying or extending their knowledge, there is some inherent ambiguity as different

> respondents will interpret the questions differently. Some will only consider only cases where they make calculations similar to those made in their classes, and others will report more advanced courses or jobs where they encountered concepts from their introductory course.

Third, we have added the following text to our conclusion:

> Educational processes are inherently complex with a myriad of decisions made by instructors and students based on a range of factors that are both observed and unobserved. The measures we use in this paper are by their nature imperfect: Test scores are incomplete measures of student knowledge, application of economic skills in future courses and jobs is subjective, and our measure of knowledge retention does not distinguish between students remembering concepts taught during the term and re-learning concepts after the term is over. Given these issues, the reader should not interpret our results as definitive. The same can be said of most educational research.

The variables used are by their nature imperfect proxies. The measures used seem reasonable – but the paper should discuss the measurement complexities. (e.g. retention v relearning, what is "using economic knowledge" in a job, what about "using economic knowledge" in other parts of life etc.?)

As stated above, we have clarified our measures of knowledge retention and application of economic knowledge in the Methods section. We have also tried our best to make this important point in our conclusion.

Editor

Before addressing the two issues raised by the editor, we would appreciate guidance on a point raised by both reviewers. In the paper, we present several findings that we believe merit consideration of possible underlying mechanisms. While the evidence is not sufficient to definitively identify these mechanisms, we offer theoretically grounded explanations that we hope can inform and motivate future empirical work. In response to reviewer feedback, we have removed speculative content that lacked a strong foundation, but we have retained the remainder of the discussion section—even though one reviewer argued for deleting the entire section. We would be grateful for your direction on this matter.

1. *In addition, you will need to discuss more carefully your sample and any factors that might affect the external validity of your findings. For example, you identify students for follow-up surveys based on students in courses with multiple students who participated in the initial assessment. Are all these classes economics courses? If not, please clarify. If so, please discuss how this may bias your results.*

   The editor is correct that the first stage of our follow-up strategy involved identifying potential students in *economics* courses that contained multiple students who participated in the initial assessment. We have clarified this in our revised draft. We have also revised our Data Description to include the following text:

   > Our follow-up success rates ranged between 25% and 46%, but it is important to consider how our data collection strategy may have biased our samples. We successfully followed up with about 10% of our target samples by surveying students in other popular economics courses, resulting in samples that likely overrepresent

students who took additional economics courses. In our second stage of follow-up, we encouraged participants by offering gift cards and explaining the value of educational research. This suggests our samples may be disproportionately composed of students who are motivated either by financial incentives or by an intrinsic interest in contributing to research. Despite these potential biases, we find that all of our follow-up samples have similar distributions of observable characteristics when compared to students in the target sample that did not participate.

2. *Furthermore, there is little information on how the courses are taught and what is emphasized in the courses. Are multiple choice exams used in the courses? Do the courses stress the use of graphs and equations or is the intro course a more issues-oriented course? More broadly, how does the fact that there is only one instructor teaching these classes affect external validity? Could this be construed as a study of the impact of one instructor's pedagogical choices?*

   We have significantly expanded our descriptions of both the introductory and intermediate-level courses by including the following text in our revised draft:

   > The [Introductory Microeconomics] course follows a traditional textbook (Krugman and Wells, 5th edition) and uses graphs and equations to define core microeconomic concepts including supply and demand, firm behavior, market structure, equilibrium, and externalities, and use them to make predictions about real world outcomes. Students are assigned 20 online problem sets during the term, and exams are closed book with a mix of multiple choice and short answer questions. The amount of active learning used in the classroom varied across the semesters as did the course modality (in-person versus online). We explore the impact of these differences in a separate paper.
   >
   > ...
   >
   > As was the case for the introductory course, the learning goals and the instructor of the intermediate course remained the same throughout the project. This is a mathematically intensive course that uses the tools of calculus, constrained optimization, and game theory to put a formal foundation under the economic concepts taught in an introductory microeconomics course. Students complete six challenging pencil-and-paper problem sets during the term, and the three major exams are composed of multipart problems that require significant mathematical calculation and deep conceptual understanding. As was the case for the introductory microeconomics course we study here, there was significant variation in the amount of active learning students did in the classroom and in the course modality.

   Reviewer 1 also wanted to see more discussion of external validity, and we have expanded our discussion with the following text:

   > A related concern is external validity. Our analysis sample is determined by the data collection design and is limited to data from six terms of two courses taught by two instructors at one institution. While Cornell University is not representative of all universities, most economics departments around the world offer courses with content that is very similar to these courses. In addition, Cornell is a selective R1

university with a student body very much like other selective R1 universities in the US. Future research could replicate this work at other institutions, with other instructors, in other courses, or even in other disciplines to determine whether long-term retention of knowledge and skills operates similarly in different contexts.

References

Adams, Wendy K. and Carl E. Wieman (2011) "Development and Validation of Instruments to Measure Learning of Expert-Like Thinking," *International Journal of Science Education*, 33 (9), 1289–1312, http://dx.doi.org/10.1080/09500693.2010.512369rm 10.1080/09500693.2010.512369.

Cosgrove, Sarah and Neal Olitsky (2015) "Knowledge Retention, Student Learning, and Blended Course Work: Evidence from Principles of Economics Courses," *Southern Economic Journal*, 82 (2), 556 – 579, http://www.jstor.org/stable/44114308.

Hestenes, David, Malcolm Wells, and Gregg Swackhamer (1992) "Force concept inventory," *The Physics Teacher*, 30 (3), 141–158, http://dx.doi.org/10.1119/1.2343497rm 10.1119/1.2343497.

Jüttler, Michael (2020) "Predicting economics student retention in higher education: The effects of students' economic competencies at the end of upper secondary school on their intention to leave their studies in economics," *PloS one*, 15 (2), e0228505, https://journals.plos.org/plosone/article?id=10.1371/journal.pone.0228505, Publisher: Public Library of Science San Francisco, CA USA.

Mulford, Douglas R. and William R. Robinson (2002) "An Inventory for Alternate Conceptions among First-Semester General Chemistry Students," *Journal of Chemical Education*, 79 (6), 739, http://dx.doi.org/10.1021/ed079p739rm 10.1021/ed079p739, Publisher: American Chemical Society.

Smith, Michelle K., William B. Wood, and Jennifer K. Knight (2008) "The Genetics Concept Assessment: A New Concept Inventory for Gauging Student Understanding of Genetics," *CBE—Life Sciences Education*, 7 (4), 422–430, http://dx.doi.org/10.1187/cbe.08-08-0045rm 10.1187/cbe.08-08-0045, Publisher: American Society for Cell Biology (lse).

Walstad, William B., Michael W. Watts, and Ken Rebeck (2007) *Test of understanding in college economics: Examiner's manual*: Council for Economic Educat.
